# *Clostridioides difficile* Enteritis Induced Anastomotic Rupture: A Case Report and Literature Review

**DOI:** 10.1155/2020/9794823

**Published:** 2020-06-13

**Authors:** David R. Velez, Mentor Ahmeti

**Affiliations:** ^1^School of Medicine & Health Sciences, Department of Surgery, University of North Dakota, 1301 N Columbia Rd Stop 9037, Grand Forks, ND 58202, USA; ^2^Sanford Medical Center, Fargo, ND, USA

## Abstract

*Background*. A 76-year-old male patient who suffered small bowel anastomotic dehiscence believed to be a complication provoked by *Clostridioides difficile* enteritis. *Case Presentation*. The patient was a 76-year-old male who underwent small bowel resection with primary anastomosis for a small bowel obstruction. On postoperative day #7, he rapidly decompensated and upon return to the operating room was found to have complete anastomotic dehiscence with copious enteric spillage. The presentation appeared as if the staple line had burst open. Enteric contents confirmed the diagnosis of *Clostridioides difficile* enteritis. Subsequent hospital course was complicated by ventilatory-dependent respiratory failure, hemodynamic instability, and persistent anemia secondary to gastric ulcer requiring endoscopic cauterization. After a prolonged hospital course, he eventually progressed and was transferred to a skilled nursing facility on hospital day #42. *Discussion*. *Clostridioides difficile* causes inflammation and copious large volume secretions that would theoretically increase intraluminal pressures creating an internal tension. This tension along with other factors from the infection itself would likely be inhibitory of anastomotic healing. Although it is rare, *Clostridioides difficile* enteritis is being reported with increasing frequency, and in the setting of recent small bowel anastomosis, it should be considered a possible risk factor for anastomotic leak.

## 1. Introduction

*Clostridioides difficile* infection (CDI) is the most common cause of health care-associated infectious diarrhea [1]. CDI has been observed to be more frequent, severe, and refractory to standard therapy than previously described [2]. This may in part be related to the epidemic strain NAP1/BI/027 which has been shown to have greatly increased toxin production, higher rates of sporulation, and increased adherence to intestinal epithelium [3]. *Clostridioides difficile* is transmitted by the ingestion of spores through a fecal-oral route. It is readily cultured in the hospital environment and has become a significant cause of morbidity and mortality among hospitalized patients [4]. Multiple risk factors commonly seen in the surgical patient have been recognized. These include antibiotic use, gastric acid suppression, gastrointestinal surgery, inflammatory bowel disease, advanced age, enteral feeding, and severe comorbid illness [1, 5, 6].

*Clostridioides difficile* is an anaerobic gram-positive bacillus that produces toxins, causing injury to intestinal epithelium leading to inflammation and diarrhea. It is able to colonize when the normal flora of the intestinal tract is disrupted. This is most commonly seen as colitis with colonization of the large intestine, although small bowel involvement with *Clostridioides difficile* enteritis (CDE) has also been described. CDE is rare; however, it is being reported in increasing frequency [7]. Although lower than first reports had indicated, mortality rates of CDE are exceptionally high, recently reported 23-30.2% [7, 8]. CDE has been poorly described in the literature and understanding of its full significance in the surgical patient is lacking. The majority of reported cases have occurred in the setting of inflammatory bowel disease (IBD) or after colonic resection [8, 9]. Specifically, the significance of CDE in the setting of recent small bowel anastomosis is unknown.

We present the case of a 76-year-old male who suffered small bowel anastomotic dehiscence believed to be a complication provoked by *Clostridioides difficile* enteritis.

## 2. Case Presentation

The patient was a 76-year-old male with past medical history significant for open cholecystectomy over ten years ago and coronary artery bypass graft less than one month prior to presentation. He was admitted for small bowel obstruction and initially treated nonoperatively. The patient failed to progress and was taken to the operating room for exploratory laparotomy with lysis of adhesions and resection of a 4.5 cm segment of ischemic small bowel with stapled anastomosis. Washout was completed and abdomen was closed.

Initial postoperative course was uneventful other than the development frequent loose stools secondary to *Clostridioides difficile* infection diagnosed by stool toxin assay on postoperative day #6. Until this point, he only had a 24-hour perioperative antibiotic coverage. He was then started on oral vancomycin with plans for discharge the next day. However, he rapidly decompensated the next morning and was found to be septic with diffuse peritonitis and spillage of enteric contents from the midline incision. The patient was emergently taken for repeat exploratory laparotomy. Upon inspection, the entire staple line of the anastomosis had dehisced and over four liters of loose enteric contents were evacuated. The proximal limb continued to briskly secrete an abnormally large amount of enteric contents. The area was further resected with new stapled anastomosis. Enteric contents obtained from the proximal end were tested and found positive for *Clostridioides difficile* toxin, confirming the diagnosis of *Clostridioides difficile* enteritis. Final pathology of small bowel resection found severe transmural ischemia.

The patient's proceeding hospital course was complicated by prolonged ventilatory-dependent respiratory failure on vasopressor support. A large fluid collection was identified with concern for possible abscess not amenable to percutaneous drainage which required surgical drainage on hospital day #18 although this fluid was found to be serous with no infection. With persistent anemia requiring multiple transfusions, he underwent endoscopy on hospital day #28 where a bleeding gastric ulcer was identified and cauterized. The patient eventually progressed and was transferred to a skilled nursing facility on hospital day #42 with physical deconditioning requiring further physical therapies. Due to continued poor oral intake and malnutrition, he underwent percutaneous endoscopic gastrostomy tube placement three days after discharge but was otherwise lost to follow-up.

## 3. Discussion

This case is remarkable for the severity and speed with which it progressed. On the verge of being discharged, just one day after the diagnosis of CDI, decompensation occurred over the course of only a few hours, and upon reentering the abdomen, the entire anastomotic staple line had dehisced with copious enteric spillage. The presentation appeared as if the staple line had burst open.

*Clostridioides difficile* causes inflammation and copious large volume secretions. Theoretically, this rise of enteric volumes would result in increased intraluminal pressures creating an internal tension. Tension has been shown to inhibit anastomotic healing [10]. This tension along with other factors from the infection itself would likely be inhibitory of anastomotic healing and place it at greater risk of leak.

A PubMed search for *Clostridioides difficile* enteritis found 274 results. These were screened by title and abstract with only available articles reviewed. Including this, 41 case reports were identified. Of these, 93% had a history of surgery involving the alimentary tract with 51% occurring within four weeks of CDE symptom onset. The average age was 55, range 20-91, with 23 females and 18 males. 54% were associated with IBD. Mortality was seen in 22%, consistent with previous reports. There were no reported cases of anastomotic leak or dehiscence.

Only two other case reports were seen in which CDE was identified in a patient who had recently undergone small bowel anastomosis [11, 12]. Both of these reports had satisfactory recovery on oral vancomycin and neither developed anastomotic leak. However, these cases did not begin to develop CDE symptoms until 3-6 weeks postoperatively, compared to less than one week as was seen here, allowing longer time for healing of the anastomotic site. There were two reported cases of ileal perforation associated with CDE, but not in the setting of small bowel anastomosis [13]. One died despite aggressive support. The other had a slow recovery but eventually discharged to a long-term care hospital.

Although CDE is rare, it is being reported more frequently and carries a high mortality. Current understanding of the full significance of CDE in the surgical patient is lacking, and further investigations are needed. However, in the setting of recent small bowel anastomosis, CDE should be considered a potential risk factor for anastomotic leak.
